# Reproduction, cultural symbolism, and online relationship: Constructing city spatial imagery on TikTok

**DOI:** 10.3389/fpsyg.2022.1080090

**Published:** 2022-12-23

**Authors:** Nuochen Liu, Xiaohui Sun, Sha Hong, Bowen Zhang

**Affiliations:** ^1^School of Advertising, Communication University of China, Beijing, China; ^2^School of Journalism and Communications, Henan University of Technology, Zhengzhou, China; ^3^College of Management, Shenzhen University, Shenzhen, China

**Keywords:** TikTok, city imagery, cultural symbolism, audience behavior, content analysis

## Abstract

The city on social media has become a hot topic in the study of city communication and city image. From the perspective of spatial theory and the communication characteristics of social media, this paper divides the spatial imagery of TikTok into three spaces: material space-cognitive attention, mental space-mental association, and relational space-emotional involvement. Based on the content analysis of 40 videos, we analyze the process of social media using cognition, association, and emotion as the starting points to increase the material space, expand the mental space, and expand the relational space. We find that spatial imagery can be co-constructed from the material space, mental space, and relational space. Lastly, the model is changed, and the value of using spatial theory to understand how city images are made is talked about.

## Introduction

TikTok enriches people’s lives with its vast information flow and ease of use, creating a convenient online communication platform between users. Users physically experience the specific space they are in, feel the people, objects, and scenery within it, and upload this process to the TikTok platform in the form of short video recordings with added topics and hashtags, which are pushed through the big data traffic pool to attract more likes, comments, and retweets from enthusiasts of the same genre, allowing more users to be present in the space (Yang et al., 2022) The location information revealed by the viewer’s work is transferred from the virtual space to the physical space, triggering secondary communication and allowing different users to form different experiences of the same urban space. Before the emergence of short-form video communication, people’s perception of a city’s image was often created by DMOs or other professional organizations that branded the city and invested heavily in relevant media, such as TV commercials, to spread the city’s image (Li Y. et al., 2020). In the current trend of convergence with the prevalence of short videos, the communication of city images is gradually changing from fixed-scale communication to personalized random communication (Wang and Feng, 2021). Because each person sees the city from a different perspective and has different interests, it enriches the city’s image on a personal level. The many personal impressions of the city provided by media platforms allow people to have a more comprehensive understanding of the city (Su, 2020b). Studies of urban spatial imagery based on social media, especially Shake, are relatively rare and limited to the use of social media data for the identification of urban spaces and images (Liu and Zhang, 2020; Ye et al., 2021), not only for the visual perception of spatial entities, but also for the attention given to the social consciousness, terroir, and cultural factors that influence the formation of imagery in cities (Gulick, 1963; Rapoport, 2016). With the development of society, the Internet, with its prominent technologies of interactivity, visualization, convenience, and multiple participation, has changed the original mechanism of city image-making. The media function has gradually shifted from recording city imagery to extensive participation in city image-making. The deep integration of social media with everyday practices, in particular, has transformed culture’s invisible into visible, establishing and creating new spatial imagery (Kong, 2020). We argue that, following the notable research of Wu et al. (2019), Kordel (2016), Gatrell and Collins-Kreiner (2006), social media users construct a spatial image of the city. This study focuses on two questions: (1) How to decompose the spatial imagery of the tourist city based on the spatial triad, and (2) How does the tourist city form a unique spatial imagery based on the communication of TikTok? This study analyses the content of city videos from four famous Chinese tourist cities (Xi’an, Changsha, Chengdu, and Chongqing) on TikTok. Based on the triadic theory of Lefebvre, the study explores the physical space, mental space, and relational space of the city through the spatial imagery constructed by TikTok. The research in this field is also made better by building a model of city spatial imagery based on TikTok.

## Literature review

### Social media and city image communication

When social media is used by public institutions, it is assessed as an innovative platform that allows these institutions to interact with citizens and other institutions (Criado et al., 2013). When social media is used by individuals, it is represented as a technology-based application that allows users to generate information and share this information (Kaplan and Haenlein, 2010). Social media, which has emerged in recent years with advances in communication technology, is shaping and changing marketing activities (Gulbahar and Yildirim, 2015), and Stankov et al. (2010) argue that they are “beginning to realize the importance of using the power of social media.” Social media is participatory, interactive, open, and transparent; it is widely used in the tourism industry, making it an appropriate way to promote cities (Zhou and Wang, 2014). Firstly, organizations (for example, destination marketing organizations, DMOs) that use social media are likely to attract the attention of more internet users (Hays et al., 2013). Secondly, opinions and advice gained through social media, especially video reviews of tourism experiences and areas, will become increasingly important in destination management decisions (Tussyadiah and Fesenmaier, 2009; Pop et al., 2022). Again, many destination management agencies are using the influence of online personalities to attract visitors to their destinations (Gretzel, 2017; Femenia-Serra and Gretzel, 2020). People are constantly using social media before, during, and after their trip to share their travel experiences (Ketter, 2016). Thus, social media has become an essential marketing strategy for tourism promotion (Chu et al., 2020; Gretzel et al. (2000); Hjalager, 2010) also mention the importance of adopting social media practices in urban tourism marketing.

Recent research has highlighted the importance of building a destination image through social media (Shao et al., 2019; Jasmin, 2020). Short video content on social media, such as microfilms, has made destinations increasingly popular (Shao et al., 2016). Through social media, cities are able to communicate with and have access to the opinions of different target audiences (Mossberger et al., 2013; Haro-de-Rosario et al., 2018). Social media has now become an optimal platform to share and improve one’s experience of visiting a city, where personal experiences and interactions are influencing people’s perceptions of the city in question (Sevin, 2014). Therefore, it would be undesirable to not make full use of social media in the marketing of a city (Gümüş, 2017).

TikTok has been studied as an effective channel for destination marketing (Li Y. et al., 2021), for example, psychological studies based on TikTok that explain why people use it (Montag et al., 2019; Ahlse et al., 2020; Marengo and Montag, 2020). TikTok fits people’s use and gratification (Katz et al., 1973; Shao and Lee, 2020), including the need for self-expression, sharing, and creation (Bucknell Bossen and Kottasz, 2020; Omar and Dequan, 2020),. In recent years, scholars have studied TikTok’s notification of health-related information (Comp et al., 2021), published official government information (Jiang and Wang, 2020), public political discussions (Medina Serrano et al., 2020), uploaded travel content (Du et al., 2020), online live sales (Su, 2020a), and broadcast educational science (Hayes et al., 2020). During the epidemic, the most recent study analyzed the video content of the TikTok platform conveying COVID-19 messages (Li X. et al., 2021), as well as the narrative approach in video epidemic reports (Sidorenko-Bautista et al., 2021). However, TikTok has been studied mainly as a marketing tool due to its huge amount of data and users, it has thus become an important platform for city marketing (Lei et al., 2020), as TikTok can make a destination famous quickly (Wengel et al., 2022). Cao et al. (2021) studied tourism behavior associated with TikTok videos; some tourists used the app for archiving, social interaction, and searching for information about attractions (Han and Zhang, 2020). Overall, the combination of TikTok and city research lies in the fact that TikTok can be used for tourism communication with short video creation and sharing (Shi, 2021), enhancing the image of cities (Li H. et al., 2020; Su, 2020a), and building city brands (Wang and Feng, 2021; Song, 2022).

### Spatial imagery of city

Boulding was the first to suggest that imagery is a condensation of people’s subjective values and knowledge, a tool for communication between subject and object, and plays an important role in the subject’s behavioral decisions (Boulding, 1956). Subsequent scholars, such as Crompton (1977) and Dichter (1985), have summarized the concept of imagery, and Lynch transposes imagery into urban studies, arguing that although residents in each city sense the city differently, it seems that each city has a common image, namely the spatial imagery of the city (Campos and Campos-Juanatey, 2019). Lynch identifies three components of an image: identity, structure, and meaning. Lynch also identified the elements of city spatial imagery, including roads, boundaries, areas, nodes, and signposts (Lynch, 1964). Many subsequent studies have adopted this classification (Young, 1999; Huang et al., 2021), and since then, the study of destinations based on city imagery has gradually become a hot topic of interest (King and Golledge, 1978; Marques et al., 2021). Although destination image research began in the 1970s, there is no clear definition in the academic community, and each scholar gives an interpretation based on their own research objectives (see Dadgostar and Isotalo, 1992; Echtner and Ritchie, 1993; Mac Kay and Fesenmaier, 2000; Bigné et al., 2001; Kim and Richardson, 2003; Tavitiyaman et al., 2021; Tasci et al., 2022). These explanations are mainly related to impressions, mental representation, and perceptual perception, which have been explored in many case studies on the process of forming destination images, the components of imagery, the factors influencing images, and the structural characteristics of imagery (Pearce, 1997; Hernández-Mogollón et al., 2018). Therefore, it can be considered that the early research on destination image began in the 1970s (Hunt, 1975; Crompton, 1979; Pearce, 1997). Researchers used the city imagery map approach to analyze the spatial imagery characteristics and patterns of the subjects (tourists) and objects (local residents) of various tourism activities in an attempt to propose a conceptual system of spatial imagery theory in the field of tourism (Pearce, 1997). In recent years, the study of spatial imagery in tourist destinations has received attention (Uusitalo, 2014; Kim et al., 2022). But the study of city images has had an effect on the study of spatial imagery in tourist destinations (Peng et al., 2020; Szubert et al., 2021), with too much focus on the spatial and structural analysis of destination cognitive maps (Jiang et al., 2009).

Walmsley and Jenkins (1992) argue that spatial imagery is the process by which people create mental images and schemas of objective objects through the recognition of their spatial characteristics. Imagery is an effective way of examining what impressions urban space leaves on visitors’ minds, and it is not just a static cognitive image in the mind; it has an impact on residents’ attitudes and behavior (Hayllar and Griffin, 2005; Amore et al., 2020), making spatial imagery an important theory and method for studying the subjective perception of urban space. Residents are both influenced and constrained by their surroundings and at the same time use them dynamically, resulting in direct or indirect experiential perceptions of their surroundings, forming a subjective environmental space in their minds (Pearce, 1999; Kitchin and Blades, 2002; Shoval, 2018; Balomenou and Garrod, 2019; Deng et al., 2021). Based on this beginning, urban spatial imagery has gradually progressed from basic research on imagery types, constituent elements, influencing factors, and formation mechanisms (Appleyard, 1970; Walmsley and Jenkins, 1992; Li and Katsumata, 2020) to the study of differences within imagery (Pacione, 2009; Koch and Latham, 2017). Studies on the spatial variability of city imagery based on socio-demographics, different social classes, and cultural differences within the city (Lathia et al., 2012; Liu and Cheng, 2020) are examples. Later on, more and more scholars began to focus on the environmental evaluation embedded in urban spatial imagery (Zhou et al., 2017) and the relationship between urban spatial imagery and citizens’ activity behavior (Chen and Tsai, 2007; Lu, 2018; Weijs-Perrée et al., 2020). Specifically, the study of urban space has evolved from a focus on residents’ perceptions to a focus on both residents and tourists (Zhao et al., 2020). In terms of research methodology, studies on the components of city spatial imagery still borrow more from Lynch’s theoretical framework (Takeuchi and Perlin, 2012), mostly using imagery sketching and spatial data analysis methods. With the advancement of technology, GIS, GPS positioning technology, and mobile phone smart navigation technology are being used as new methods to obtain spatial data from tourists (Xia et al., 2008; Salas-Olmedo et al., 2018; Vaez et al., 2020).

In the field of city spatial imagery research, the hotspots are clustered in four areas: (1) the characteristics of urban spatial imagery as determined by a dynamic and comparative data analysis (Huang, 2002; Kitchin and Blades, 2002). (2) The effects of city spatial imagery, which is thought to influence residents’ attitudes and behaviors (Hayllar and Griffin, 2005), such as influencing residents’ evaluation of their activity experience (Smith and McGillivray, 2022), evaluation of the urban environment (Abass et al., 2019), behavioral choices (Prayag et al., 2017), attitudes towards urban resource management measures (Ramkissoon and Nunkoo, 2011; Hammitt et al., 2015), and urban image formation and communication (Bavinton, 2013; Hernández-Mogollón et al., 2018). (3) The formation mechanism of city spatial imagery; investigating the various influencing factors and causes of city spatial imagery; and comprehending the inner formation mechanism of city spatial imagery. The city environment and the residents are the primary influencing factors of city spatial imagery (Hunt, 1971; Pike, 2002). City environmental factors mainly refer to spatial structure (Pearce, 1998; Smith, 2005), city culture and urban construction (Appleyard, 1970), spatial and temporal distance, cultural differences, and economic level. The factors affecting city residents themselves mainly include gender and age; economy; experience; social class; education level; and value system (Peel and Lloyd, 2008; Bonakdar and Audirac, 2020). In addition, city spatial imagery is influenced by city events (Lee et al., 2005; Jeong and Kim, 2019); advertising (Peel and Lloyd, 2008; Wang and Feng, 2021); marketing (Hospers, 2009; Deffner et al., 2020); and media (Stepchenkova and Eales, 2011; Li et al., 2018). (4) The creation of city spatial imagery is currently being analyzed from the perspective of the interaction between residents and the city environment, using imagery space and its composition to analyze the problems in the development of urban space (Zhang et al., 2009; Chen et al., 2022). Most of the studies, from a research point of view, have been done from the field of communication studies, using mathematical and statistical methods to look at the issues at hand. However, there aren’t many case studies that look at how the audience perceives space.

Lynch’s approach to city spatial imagery was limited by the time period, as the investigation process needed to be based on the subject’s personal experience of the physical urban space (Lee and Schmidt, 1986; Hátlová and Hanus, 2020), and Guy et al.’s (1990) study of Wurzburg, Germany, showed that field experiences had the most profound impact on the spatial cognitive processes of tourists. Previous studies on spatial imagery in urban tourism have thus concentrated on the city’s physical tourism space, employing more tourism cognitive maps and interview research methods. However, there are also convergences and innovations, and technical approaches from different disciplines such as geography, sociology, and psychology have been effectively integrated into the study of city spatial imagery (Echtner and Ritchie, 1993; Son, 2005; Oteros-Rozas et al., 2018; Lalicic et al., 2021). In recent years, there have been a number of examples of the use of mass media such as television and film to study city spatial imagery (Massood, 2011; Wen et al., 2018). As online technologies have penetrated the media landscape, online media have gradually become a platform for urban tourism research, but research based on online texts has mostly focused on the perception of city images (Pikkemaat, 2004; Choi et al., 2007; Sugandini, 2020; Chen et al., 2022) and rarely extended to the study of spatial imagery in urban tourism. This paper conducts a study of spatial imagery in cities based on online content, i.e., the videos and texts of TikTok. It not only uses the method of studying spatial imagery with online content, but it also adds to the research on how people see spatial images of cities.

## Research method

### Case selection and data origin

The selection of the case was divided into two steps: the selection of the case city and the selection of the city’s TikTok videos.

In the first step, TikTok China was selected as the short video platform for this study. TikTok China was searched for the names of 34 cities in the list of “Excellent Tourism Cities in China” published by the China National Tourism Administration (see Appendix 3). The 34 cities were then ranked by the total number of videos, likes, and favorites on the TikTok China platform, and the top four cities were selected: Xi’an, Changsha, Chengdu, and Chongqing.

In the second step, the top 10 videos from the four cities were ranked in descending order by the number of likes for further video selection. The top 10 videos in terms of the number of likes were selected, and the main contents of the videos could not be duplicated, so that the famous places in each city were ranked according to the number of likes. Appendix 2 presents a detailed list of the famous tourism sites that appear in the TikTok videos for each of the four cities and the number of times they appear. After obtaining the names of tourism sites in each city’s TikTok videos, we searched for videos by searching for the keywords “city name + tourism site name” in April 2022 and obtained the top 10 videos with the highest number of likes in each of the four cities. In total, 40 videos were obtained.

### Data analysis process

**Figure 1:**
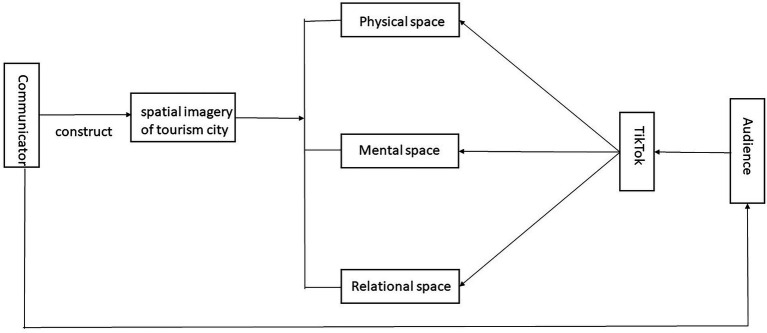
The physical, mental, and relational imagery of a tourism city.

In this study, all videos were open-coded using a qualitative content analysis approach. Following the theory of Lefebvre (2003, 2012) and the characteristics of the research object of study, the conceptual model was constructed (see Figure 1). Based on this model and the coded content, the study analyses the physical, mental, and relational dimensions of city spatial imagery. The research question is answered by exploring and answering the following three sub-questions: Is there a before and after change in the physical dimension of the tourist space in the video? Is there a new mental symbolism in the tourist space? Does the tourist space have any realistic relevance to a wider range of people? The role that TikTok played in these three stages of change is also looked at in the context of how TikTok works as a media platform.

## Research findings

Based on the aforementioned data selection, this study identified the following four cities for their main video content. The famous hit spots in **Xi’an** city include Bell and Drum Tower, Xi’an City Wall, The Great Tang Dynasty City of Night, Big Wild Goose Pagoda, and Terracotta Warriors; the famous hit spots in **Changsha** city include Super Wenheyou, International Finance Centre (IFC), Orange Island, Yuelu Mountain, and May Day Square; the famous hit spots in **Chongqing** city include Liziba Light Rail, Hongya Cave, Yangtze River Ropeway, Kui Xing Lou, White Elephant House, and Chuan Mei Graffiti Street; and **Chengdu** city include Chunxi Road, Kuanzhai Alley, Jinli, Panda Base, and Wuhou Temple. We can see that the number of likes on some of these videos is in the hundreds of thousands or even millions, and the number of favorites, comments, and retweets is also high. The city’s famous spots are popular on TikTok (Zihan, 2019; Dexing, 2020).

### Changing the physical dimension of the city by drawing the audience’s cognitive attention

Physical space is the physical presence of spatial imagery, which can be accurately measured, depicted, and designed with the help of instruments and tools within a certain range, and its corresponding space has a physical form, a dimension that can be perceived by touch (Wu, 2011). The physical space of a city refers to the architectural design, street layout, and texture of its landscape and involves the possibility and convenience of human mobility and interaction (Sun, 2018).

The physical spaces are mainly analyzed through the video content (see Appendix 1 Pictures 1–6). Appendix 1 Picture 1 is the Xi’an City Wall, the largest city wall remaining with over 1,600 years of history; Appendix 1 Picture 2 is Orange Island, a famous political site in China; Appendix 1 Picture 3 is Hongya Cave, which features a gloriously lit and strange landscape; Lulu (2021) is the Datang City of Night; Appendix 1 Pictures 5 and 6 are the Taikoo Li naked eye 3D screen and the Li Ziba light rail tram, which feature visual stimulation. The reason for the high popularity of these videos is twofold: on the one hand, it is that the spaces of the hit places in the videos, as shown in Appendix 1 Pictures 1–6, all have the characteristics of physical space that attract tourists, and they all have the characteristics of visual stimulation, such as being large and strange at the level of physical form. The video production, on the other hand, fits the “short, fresh, and fast” characteristics of the short video platform (Shen, 2019), which quickly pushes visually impactful images to the users’ eyes in a short period of time, stimulating their visual senses and mapping the physical form of the city; at the same time, the selection of music or accompanying text that has recently been popular on social media. The campaign also used songs or texts that have been popular on social media lately, which brought more people to the internet.

Of the TOP 100 city promotion videos played on the TikTok platform, “more than 80% were created by individual users” (TikTok Headline Index, Urban Branding Research Office, Tsinghua University, 2018). In these videos, tourists evaluate the original physical space on the basis of their existing perceptions, and the features that are unearthed or discovered are edited by tourists into informative content and uploaded to social media. For example, Li (2018, 2022) shows a visitor’s uploaded carriage of the Chongqing Yangtze River Ropeway scenic spot. After gaining a high level of cognitive attention on TikTok, the carriage of the Chongqing Yangtze River Ropeway scenic spot is then replaced with a new paint job in a video later uploaded by the visitor. Another example is that after Liziba Metro Station gained online attention on TikTok, the local managers deliberately added a viewing platform to meet the demand of tourists to take and make photographs (see Picture 7 in the Appendix 1). As a result of the influence of social media, the local authorities will transform the physical space according to the popularity of the spot. To keep up with the popularity of the videos, the Changsha government made special pink zebra crossings for a local festival and heart-shaped traffic lights for Valentine’s Day (see Picture 8 in the Appendix 1) to match the festive atmosphere.

The distinctive physical forms paired with the techniques of TikTok video production can quickly draw attention to the TikTok platform’s traffic recommendation, sharing, and forwarding functions, and the physical spaces attract more attention and expand audience awareness (both resident and tourist) of these physical spaces due to the fission spread of social media. The managers transformed the spatial cities according to the popularity of the TikTok videos and the needs of the visitors, and the local tourism management department played the role of active transformation while making reactive adjustments. This is a new phenomenon in China’s urban renewal process, which validates that the creation of internet influencers is not only limited to individuals but is transforming urban physical spaces (Li Y. et al., 2020). Whether the local government is passively changing or actively making new things, it shows that physical spaces are always being made better because of the attention they get on social media.

### Enlarging the mental dimension of the city by triggering psychological associations

The representation of mental space is the conceptualization of knowledge, symbols, and order by scientists, planners, urbanists, and others, and its counterpart is the space occupied by sensory phenomena, which is a constructed dimension (Wu, 2011). TikTok’s video content comes from how visitors choose to filter and edit their videos. This makes TikTok’s video content a “consciously processed space” that shows the real physical qualities of space and gives it a mental dimension because of how people process it.

The formation of a mental dimension is twofold. Firstly, there is the generation of spiritual symbols in space. Every TikTok user who registers an account has the power to post videos and is then entitled to participate directly in the construction and dissemination of urban space. For example, Jinli is a quaint stone street and riverside pavilion next to the Wuhou Temple to Chengdu residents, but in TikTok’s, Jinlin is not only about snacks, an ancient theatre, and the Adu Well, but also the “First Street of Western Sichuan,” the “Chengdu version of Qingming Shanghe Tu,” and the “Three Kingdoms Culture” (hashtags in TikTok videos). Through the spread of TikTok, these cultural symbols arouse the audience’s associations and are integrated into the construction of the city’s spatial image (see Picture 9 in the Appendix 1). Chengdu Heming Tea House became a new TikTok hot spot in May 2020, following the end of the city quarantine. The tea house has become a space for boat cruisers, tea drinkers, card players, and ear pickers, as well as a window for cultural exchange and spiritual interaction between locals and tourists from all over the world. While satisfying cultural and spiritual needs, they have also created new spiritual symbols. It is worth noting that nearly half of the videos in the TOP 100 on the TikTok platform contain the challenge hashtag (see example in Appendix 1; TikTok Headline Index, Urban Branding Research Office, Tsinghua University, 2018). As shown in the red box circled in the example in Zhihong (2020, 2021), multiple users create videos around the same topic, and short videos with added challenge tags receive better recommendation priority. Through this challenge, the video platform can get more users to make videos and reach a wider audience. This will help tap into new symbols and give the space more spiritual and symbolic symbols (Zhihong, 2020, 2021).

Secondly, there are interpretations of mental symbols in space. Although the reader can produce a concretized reading from different perspectives, what is ultimately left in the mind is not a specific text or a complete part of a text but a condensed gestalt that remains in the viewer’s mind that can correspond to spatial forms and play with subjective associations (Kauffka, 1997). Archetypal imagery is physical and has trans-subjective qualities, and the reader is able to respond to its materiality and qualities in the same way, such as the warm and restful feeling of house imagery (Gaston, 1964). In Appendix 1 Picture 11, the video shows the “forever street” of Changsha Super Wenheyou, the childhood kiosk, the lay-off brand of stinky tofu and the alleyway pig’s feet, leading to the “old Changsha,” “eighties,” “three-dimensional scene-based food magic formula,” “Taste of Changsha” and other symbolic meanings from Chinese literature and films related to the city, reinforcing the spatial imagery of Super Wenheyou. And combined with the comments shown in Appendix 1 Picture 12, we can see that in TikTok’s video, these symbols are naturally able to decode the spiritual symbols conveyed in the video and associate with the spiritual space of the tourist destination as they are edited and presented in a modern discourse. In addition, in Appendix 1 Picture 13, the spaces are tagged during the display process and are constantly mentioned through the TikTok platform, so that audiences will associate the corresponding space when they see the video tag, for example, the tag “Chongqing 8D Magic Space” which corresponds to the space of the Li Ziba light rail station where the train passes through the building, the Cathay Art Centre, which is made up of several three-dimensional chopsticks stacked on top of each other, and the road like waves in Chongqing city.

The original contents posted by individual TikTok users and the associations based on films, music, literature, and other texts can add new spiritual and cultural symbols to the physical space; the participants are not limited to local residents who have already formed a stereotype of the city but also include tourists with different purposes of travel. The different content and styles of photography that different tourists focus on continue to trigger mental associations and thus generate spiritual symbols; the challenge of the hashtag motivates more users to associate with the same space, leading to the creation of spiritual symbols. Thus, the mental space is continually replenished and expanded. The modern style of the story and the way that the labels correspond to the space make it possible to figure out what the spiritual symbols in the space mean and how they relate to the physical space.

### Expanding the relational space of the city by guiding the audience’s emotional involvement

Relational space is the overarching dimension of social relations, co-created by the participants in spatial practices, and its counterpart, the social space, is the dimension in which people live (Wu, 2011). People’s relationship to the past also includes a connection to space (Stephanson and Jameson, 1989).

In Pictures 14 and 15 of Appendix 1, the transformation of the phenomenon of fan-circleisation (the online gathering of fans) into a real space is illustrated. The video in Appendix 1 Picture 14 shows the filming location of a Chinese film, and most of the commenters on the video are fans of the film, with the comment section gathering a large number of fans of the male protagonist who say they would “love to go to the filming location in person.” So in Appendix 1 Picture 15, we can see the fan groups gathering at Chongqing’s Railway High School, Kui Xing Lou, Crown Escalator, and Zhongshan 4th Road to take photos and make these spaces interactive with people, creating a relationship between space and people. In Appendix 1 Picture 16, Xie Xiaojiu, the ‘Master of Nine’ from Xi’an, sings about the city and attracts many folk singers to join him. Based on the emotional identity of the music, residents and tourists came to and performed at the South Gate of the City Walls in Xi’an City. Wang and Xu (2021) say that the new Internet business model in China tries to get and control huge amounts of data to understand how users feel and what they want. Based on big data recommendations, TikTok suggests these video contents to users who like the film, the protagonists, or certain topics. This increases the number of people who follow this video, which can be turned into more offline experiences.

According to the narrative, the space on TikTok can be divided into story space and discursive space. The former refers to the present context in which the act or story takes place, while the latter refers to the narrator’s space, including the context in which the narrator tells or writes (Herman et al., 2010). Most of the videos are dominated by the narratives of TikTok users, and the narrator is often the experiencer of the space. In Lulu (2021), TikTok users act as narrators, using real punchline photos and narration to create a sense of authenticity. The relaxed, chatty video copy quickly closes the distance between the viewer and the audience, gaining likes and retweets. This authentic and intimate approach brings the viewer closer, creating an emotion of trust that makes the viewer yearn for the punching place as well. Social media’s ability to make people feel like they are there can also be very evocative. This happens when the setting of the act or story overlaps with the narrator’s space, creating a great sense of immersion and making people want to go there.

The algorithm-based TikTok platform accelerates and amplifies emotional relationships online, enabling the transformation of real space. The stories in the videos are based on the real-life experiences of the narrators. They are paired with intimate chatter that creates a sense of being there and can make viewers feel connected to the space in the video.

## Conclusion and discussion

**Figure 2:**
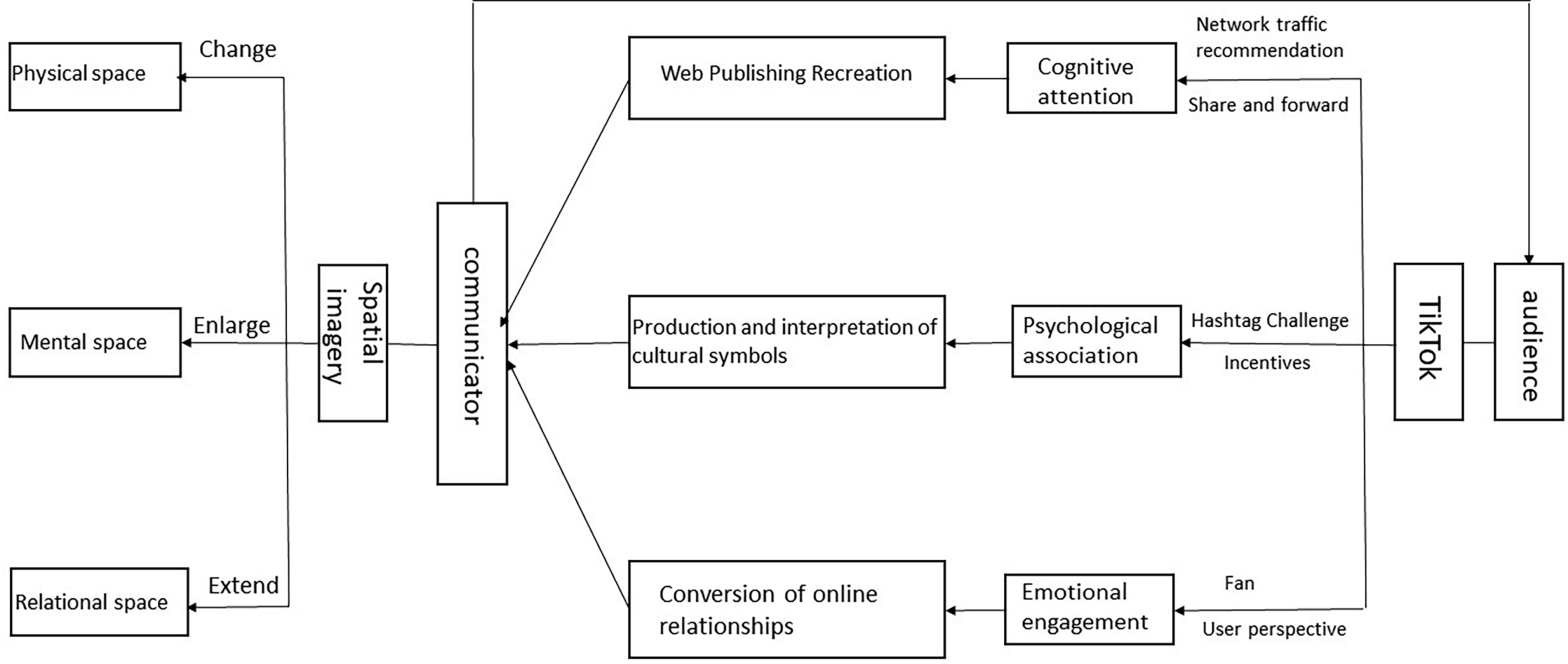
Physical, mental, and relational imagery of city on TikTok (PMRT model).

The first aim of this study is to explore how to decompose the shaping of spatial imagery in tourist cities. Several studies have been conducted in the past (see Gatrell and Collins-Kreiner, 2006; Kordel, 2016; Wu et al., 2019), but the case in this study focuses on the social media (TikTok) platform to analyze how tourist cities have formed unique spatial imagery based on the communication of TikTok. This is also the second aim of this study: to explore the model and ways of shaping spatial imagery based on the communication of the TikTok. Previous studies have demonstrated that social media can be a communication channel for tourism promotion and city image enhancement in tourist cities (Hjalager, 2010; Zhou and Wang, 2014). The results of this study show that TikTok can also effectively contribute to the construction of spatial imagery in cities through three dimensions: physical, mental, and social relations. At the physical level, the study demonstrates the importance of the cognitive attention that TikTok can trigger, as characteristic spatial forms are quickly recognized in the TikTok medium and provoke people to attend, so that those who visit will recreate the spatial forms and post them on the TikTok platform, and the recreated works will attract official attention for the augmentation of physical spaces in the city. TikTok, on a mental level, becomes a platform for tourist cities to discover spiritual symbols such as history and culture, and its incentives encourage users to combine texts such as music, literature, film, and television with relevant tourist cities, thereby continuously expanding the spiritual connotations in spatial imagery. The result of text analysis in the video supports the idea that an increase in spiritual symbols will help people identify more with this city. At a relational level, TikTok supports the presence of the fan community (Fathallah, 2020; Lynch, 2022), and the tourist city will touch on an element of fan culture that triggers a large number of people to interact with the space, and the research confirms that emotions can be the link that expands the relational space. TikTok therefore plays a mediating role in all three dimensions of space: physical, mental, and relational, thus assisting in the construction of spatial imagery in the tourist city. Furthermore, the findings confirm that tourist cities with a deep historical and cultural heritage have a great advantage in the production of spiritual space, such as the Tang Dynasty culture in Xi’an. The unique cultural elements in a city are spiritual symbols that are not replicable, and this study therefore argues that tapping into irreproducible spiritual cultural symbols and shaping irreproducible spatial imagery is crucial for the communication of tourist cities. Based on the aforementioned content analysis and the above findings, this study improves the conceptual model (see Figure 2) to better apply to the analysis of city spatial imagery on TikTok.

### Beyond the spatial dichotomy: The subject-object cycle in the three dimensions of space imagery

This study not only adds to the study of spatial imagery in tourist cities and related spaces but also makes three contributions to research in related fields. Firstly, this study constructs a model (see Figure 2) by introducing the mediating role of TikTok, which empowers a way of thinking about the construction of spatial imagery in tourist cities. The logic in the model clarifies which features of social media are utilized and which landing points can interfere with the three dimensions of spatial imagery. It extends the perspective of constructing spatial imagery through organizations or local authorities (Ramkissoon and Nunkoo, 2011; Hammitt et al., 2015) by highlighting the role of the social media user (the audience) in the construction of spatial imagery. The second contribution is that the findings show a cyclical construction of the communicator and audience in the three dimensions of spatial imagery. While the communicator (here referring to the organization or agency that constructs and communicates the city image) constructs spatial imagery through physical, mental, and relational imagery, the audience is able to participate in each level of spatial imagery construction through the media advantage of TikTok, thus influencing the city managers’ adjustment of spatial imagery. When the adjusted spatial imagery is disseminated through TikTok, the actions of the audience in turn influence the construction of spatial imagery in the tourist city. Thirdly, the formation of spatial imagery in this study confirms that space (the city) is no longer a dichotomy between physical and mental (Milevsky and Moshe, 1997; Bystrowska and Dolnicki, 2017), that the spiritual symbols of space are dependent on the forms of physical space, and that relational space arises as a fusion of physical space and spiritual space. Also, the existence of relational space affects the physical forms and spiritual contents of the space, which in turn affects spatial imagery, and the continued existence of relational space ensures that spatial imagery will last.

### Shaping spatial imagery based on the PMRT model: The practical implications

The PMRT model can serve as a conceptual and operational guide for tourism city managers looking to shape their city’s image. Faced with a competitive tourism industry and diverse communication channels, tourism city managers have also been exploring how to better promote and publicize their cities. The shaping of spatial imagery realizes the psychological link between the city and people, so that the image of the tourist city does not only stay at the level of material forms but can communicate and exchange with people in spirit, thus promoting the interaction between people and the tourist city and the relationship with this tourist city. The PMRT model points out that city images can be constructed from three dimensions of city imagery: physical space, mental space, and relational space based on cognitive attention, mental association, and emotional engagement (see Figure 2). Specifically, through TikTok’s traffic recommendation, commenting and sharing, users re-create post contents; through tag challenges and incentive mechanisms, produce and interpret urban cultural symbols; and based on their own perspectives and fan group activities, they realize the transformation of online relationships.

Although the role played by city managers in city image communication is crucial (Booms and Bitner, 1980; Krupskyi and Grynko, 2018), the PMRT model highlights the importance of the audience, which includes both residents and tourists. And it advocates the need to think about residents in city image communication (Braun et al., 2013) and the power of the social media user (Sevin, 2014; Wang and Feng, 2021). The process of constructing city images can make full use of the advantages of social media to realize the co-creation of city imagery.

### Limitation and future research

We must acknowledge that the study has certain limitations. Firstly, this study was conducted in a single setting, i.e., it was limited to Chinese cities. Future studies should therefore go further to validate the model and apply it to cities worldwide in order to generalize the findings. The limitations of the study, in addition, are its context and generalizability outside of China, as it is quite a different market from the rest of the world. It is important to note that the case data collection for this study was limited to China. Therefore, it is worthwhile to conduct a cross-cultural study. Furthermore, TikTok is only one type of social media platform, and although it is extremely representative, it is still important to conduct validation of other social media types if the study is to be applied to social media. This is also a direction for future research.

## Data availability statement

The original contributions presented in the study are included in the article/Supplementary material, further inquiries can be directed to the corresponding author.

## Author contributions

NL and BZ contributed equally to this work and share the first authorship. NL and BZ contribute to the design and implementation of the research; XS and SH collect the data and conduct analysis of the results. All author contribute to the writing of the manuscript.

## Conflict of interest

The authors declare that the research was conducted in the absence of any commercial or financial relationships that could be construed as a potential conflict of interest.

## Publisher’s note

All claims expressed in this article are solely those of the authors and do not necessarily represent those of their affiliated organizations, or those of the publisher, the editors and the reviewers. Any product that may be evaluated in this article, or claim that may be made by its manufacturer, is not guaranteed or endorsed by the publisher.

## Supplementary material

The Supplementary material for this article can be found online at: https://www.frontiersin.org/articles/10.3389/fpsyg.2022.1080090/full#supplementary-material

## References

[ref1] Abass K. Appiah D. O. Afriyie K. (2019). Does green space matter? Public knowledge and attitude towards urban greenery in Ghana. Urban For. Urban Green. 46:126462. doi: 10.1016/j.ufug.2019.126462

[ref2] Ahlse J. Nilsson F. Sandström N. (2020). It’s time to tiktok: exploring generation z’s motivations to participate in #challenges. Available at: http://urn.kb.se/resolve?urn=urn:nbn:se:hj:diva-48708 (Accessed November 10, 2022).

[ref3] Amore A. Falk M. Adie B. A. (2020). One visitor too many: assessing the degree of overtourism in established European urban destinations. Int. J. Tour. Cities. 6, 117–137. doi: 10.1108/IJTC-09-2019-0152

[ref4] Appleyard D. (1970). Styles and methods of structuring a city. Environ. Behav. 2, 100–117. doi: 10.1177/001391657000200106

[ref5] Balomenou N. Garrod B. (2019). Photographs in tourism research: prejudice, power, performance and participant-generated images. Tour. Manag. 70, 201–217. doi: 10.1016/j.tourman.2018.08.014

[ref6] Bavinton N. (2013). “Putting leisure to work: city image and representations of nightlife,” in Culture and the City. (London: Routledge), 49–63.

[ref7] Bigné J. E. Sánchez M. I. Sánchez J. (2001). Tourism image, evaluation variables and after purchase behaviour: inter-relationship. Tour. Manag. 22, 607–616. doi: 10.1016/S0261-5177(01)00035-8

[ref8] Bonakdar A. Audirac I. (2020). City branding and the link to urban planning: theories, practices, and challenges. J. Plan. Lit. 35, 147–160. doi: 10.1177/0885412219878879

[ref9] Booms B. H. Bitner M. J. (1980). New management tools for the successful tourism manager. Ann. Tour. Res. 7, 337–352. doi: 10.1016/0160-7383(80)90027-4

[ref10] Boulding K. E. (1956). The image: knowledge in life and society. Rexdale, Canada: University of Michigan Press, 10–11.

[ref11] Braun E. Kavaratzis M. Zenker S. (2013). My city-my brand: the different roles of residents in place branding. J. Place Manag. Dev. 6, 18–28. doi: 10.1108/17538331311306087

[ref12] Bucknell Bossen C. Kottasz R. (2020). Uses and gratifications sought by pre-adolescent and adolescent Tik Tok consumers. Young Consum. 21, 463–478. doi: 10.1108/YC-07-2020-1186

[ref13] Bystrowska M. Dolnicki P. (2017). The impact of endogenous factors on diversification of tourism space in the Arctic. Curr. Issue Tour. 5, 36–43.

[ref14] Campos A. Campos-Juanatey D. (2019). The representation of imagery of the city: the impact of studies and imagery ability. Jpn. Psychol. Res. 61, 179–191. doi: 10.1111/jpr.12208

[ref15] Cao X. Qu Z. Liu Y. Hu J. (2021). How the destination short video affects the customers’ attitude: the role of narrative transportation. J. Retail. Consum. Serv. 62:102672. doi: 10.1016/j.jretconser.2021.102672

[ref16] Chen C. Li H. Luo W. Xie J. Yao J. Wu L.. (2022). Predicting the effect of street environment on residents’ mood states in large urban areas using machine learning and street view images. Sci. Total Environ. 816:151605. doi: 10.1016/j.scitotenv.2021.151605, PMID: 34838562

[ref17] Chen C.-F. Tsai D. (2007). How destination image and evaluative factors affect behavioral intentions? Tour. Manag. 28, 1115–1122. doi: 10.1016/j.tourman.2006.07.007

[ref18] Choi S. Lehto X. Y. Morrison A. M. (2007). Destination image representation on the web: content analysis of Macau travel related websites. Tour. Manag. 28, 118–129. doi: 10.1016/j.tourman.2006.03.002

[ref19] Chu S.-C. Deng T. Cheng H. (2020). The role of social media advertising in hospitality, tourism and travel: a literature review and research agenda. Int. J. Contemp. Hosp. Manag. 32, 3419–3438. doi: 10.1108/IJCHM-05-2020-0480

[ref20] Comp G. Dyer S. Gottlieb M. (2021). Is Tik Tok the next social media frontier for medicine? AEM Educ. Train. 5:10532. doi: 10.1002/aet2.10532, PMID: 34095694 PMC8155692

[ref21] Criado J. I. Sandoval-Almazan R. Gil-Garcia J. R. (2013). Government innovation through social media. Gov. Inf. Q. 30, 319–326. doi: 10.1016/j.giq.2013.10.003

[ref22] Crompton J. L. (1977). A systems model of the tourist’s destination selection decision process with particular reference to the role of image and perceived constraints, vol. Volumes I and II. Texas: Texas A and M University.

[ref23] Crompton J. L. (1979). An assessment of the image of Mexico as a vacation destination and the influence of geographical location upon that image. J. Travel Res. 17, 18–23. doi: 10.1177/004728757901700404

[ref24] Dadgostar B. Isotalo R. M. (1992). Factors affecting time spent by near-home tourists in City destinations. J. Travel Res. 31, 34–39. doi: 10.1177/004728759203100206

[ref25] Deffner A. Karachalis N. Psatha E. Metaxas T. Sirakoulis K. (2020). City marketing and planning in two Greek cities: plurality or constraints? Eur. Plan. Stud. 28, 1333–1354. doi: 10.1080/09654313.2019.1701291

[ref26] Deng Z. Chen D. Qin X. Wang S. (2021). Comprehensive assessment to residents’ perceptions to historic urban center in megacity: a case study of Yuexiu District, Guangzhou, China. J. Asian Archit. Build. Eng. 20, 566–580. doi: 10.1080/13467581.2021.1942000

[ref270] Dexing Li. (2020). [@cover News]. [Video]. Available at: https://v.douyin.com/rQdqL92/ (Accessed December 25, 2020).

[ref27] Dichter E. (1985). What’s in an image. J. Consum. Mark. 2, 75–81. doi: 10.1108/eb038824

[ref28] Du X. Liechty T. Santos C. A. Park J. (2020). ‘I want to record and share my wonderful journey’: Chinese Millennials’ production and sharing of short-form travel videos on Tik Tok or Douyin. Curr. Issue Tour. 25, 3412–3424. doi: 10.1080/13683500.2020.1810212

[ref29] Echtner C. M. Ritchie J. R. B. (1993). The measurement of destination image: an empirical assessment. J. Travel Res. 31, 3–13. doi: 10.1177/004728759303100402

[ref30] Fathallah J. (2020). Digital fanfic in negotiation: live journal, archive of our own, and the affordances of read–write platforms. Convergence 26, 857–873. doi: 10.1177/135485651880667

[ref31] Femenia-Serra F. Gretzel U. (2020). “Influencer marketing for tourism destinations: lessons from a mature destination,” in Information and communication Technologies in Tourism. 2020. eds. Neidhardt J. Wörndl W. (Cham: Springer), 65–78.

[ref32] Gaston Bachelard. (1964). The poetics of space. New York: The Orion Press.

[ref33] Gatrell J. D. Collins-Kreiner N. (2006). Negotiated space: tourists, pilgrims, and the Bahá’í terraced gardens in Haifa. Geoforum 37, 765–778. doi: 10.1016/j.geoforum.2006.01.002

[ref34] Gretzel U. (2017). Influencer marketing in travel and tourism. Advances in social media for travel, tourism and hospitality. London: Routledge, 147–156.

[ref35] Gretzel U. Yuan Y.-L. Fesenmaier D. R. (2000). Preparing for the new economy: advertising strategies and change in destination marketing organizations. J. Travel Res. 39, 146–156. doi: 10.1177/004728750003900204

[ref36] Gulbahar M. O. Yildirim F. (2015). Marketing efforts related to social media channels and Mobile application usage in tourism: case study in Istanbul. Proc.-Soc. Behav. Sci. 195, 453–462. doi: 10.1016/j.sbspro.2015.06.489

[ref37] Gulick J. (1963). Images of an Arab city. J. Am. Inst. Plann. 29, 179–198. doi: 10.1080/01944366308978063

[ref38] Gümüş N. (2017). Usage of social media in city marketing: a research on 30 metropolitan municipalities in Turkey. EMAJ Emerg. Mark. J. 6, 30–37. doi: 10.5195/EMAJ.2016.114

[ref39] Guy B. S. Curtis W. W. Crotts J. C. (1990). Environmental learning of first-time travelers. Ann. Tour. Res. 17, 419–431. doi: 10.1016/0160-7383(90)90007-E

[ref40] Hammitt W. E. Cole D. N. Monz C. A. (2015). Monz. Wildland recreation: Ecology and management. Oxford: John Wiley & Sons.

[ref41] Han M. Zhang X. (2020). “Prospects for the advancement of the Tik Tok in the age of 5G communication.” in: *2020 13th CMI conference on Cybersecurity and privacy (CMI) - digital transformation-potentials and challenges (51275)*. IEEE, Copenhagen, Denmark, 1–5.

[ref42] Haro-de-Rosario A. Sáez-Martín A. del Carmen Caba-Pérez M. (2018). Using social media to enhance citizen engagement with local government: twitter or Facebook? New Media Soc. 20, 29–49. doi: 10.1177/1461444816645652

[ref43] Hátlová K. Hanus M. (2020). A systematic review into factors influencing sketch map quality. ISPRS Int. J. Geo-Inf. 9:271. doi: 10.3390/ijgi9040271

[ref44] Hayes C. Stott K. Lamb K. J. Hurst G. A. (2020). “Making every second count”: utilizing Tik Tok and systems thinking to facilitate scientific public engagement and contextualization of chemistry at home. J. Chem. Educ. 97, 3858–3866. doi: 10.1021/acs.jchemed.0c00511

[ref45] Hayllar B. Griffin T. (2005). The precinct experience: a phenomenological approach. Tour. Manag. 26, 517–528. doi: 10.1016/j.tourman.2004.03.011

[ref46] Hays S. Page S. J. Buhalis D. (2013). Social media as a destination marketing tool: its use by national tourism organisations. Curr. Issue Tour. 16, 211–239. doi: 10.1080/13683500.2012.662215

[ref47] Herman D. Jahn M. Ryan M. L. (2010). Routledge encyclopedia of narrative theory. London: Routledge.

[ref48] Hernández-Mogollón J. M. Duarte P. A. Folgado-Fernández J. A. (2018). The contribution of cultural events to the formation of the cognitive and affective images of a tourist destination. J. Destin. Mark. Manag. 8, 170–178. doi: 10.1016/j.jdmm.2017.03.004

[ref49] Hjalager A.-M. (2010). A review of innovation research in tourism. Tour. Manag. 31, 1–12. doi: 10.1016/j.tourman.2009.08.012

[ref50] Hospers G.-J. (2009). Lynch, Urry and city marketing: taking advantage of the city as a built and graphic image. Place Brand. Public Dipl. 5, 226–233. doi: 10.1057/pb.2009.10

[ref51] Huang Y. P. (2002). Urban space theory and spatial analysis. Fujian: Southeast University Press.

[ref52] Huang J. Obracht-Prondzynska H. Kamrowska-Zaluska D. Sun Y. Li L. (2021). The image of the city on social media: a comparative study using “big data” and “small data” methods in the tri-city region in Poland. Landsc. Urban Plan. 206:103977. doi: 10.1016/j.landurbplan.2020.103977

[ref53] Hunt J. D. (1971). Image: A factor in tourism. Fort Collins: Colorado State University.

[ref54] Hunt J. D. (1975). Image as a factor in tourism development. J. Travel Res. 13, 1–7. doi: 10.1177/004728757501300301.41

[ref55] Jasmin A. S. (2020). Pose strategy as a media for tourism destination promotion in Batu city, East Java. J. Indones. Tour. Policy Stud. 5, 33–47. doi: 10.7454/jitps.v5i1.173

[ref56] Jeong Y. Kim S. (2019). A study of event quality, destination image, perceived value, tourist satisfaction, and destination loyalty among sport tourists. Asia Pac. J. Mark. Logist. 32, 940–960. doi: 10.1108/APJML-02-2019-0101

[ref57] Jiang J. Wang W. (2020). Research on government Douyin for public opinion of public emergencies: comparison with government microblog. J. Intel. 39, 100–106.

[ref58] Jiang Z. Zhang J. Han G. Cao J. (2009). A study review of cognitive maps of tourists. Tour. Trib. 24, 77–85.

[ref59] Kaplan A. M. Haenlein M. (2010). Users of the world, unite! The challenges and opportunities of social media. Bus. Horiz. 53, 59–68. doi: 10.1016/j.bushor.2009.09.003

[ref60] Katz E. Blumler J. G. Gurevitch M. (1973). Uses and gratifications research. Public Opin. Q. 37, 509–523. doi: 10.2307/2747854

[ref61] Kauffka K. (1997). Principles of gestalt psychology, translated by Lai Wei, Hangzhou: Zhejiang Education Press, 46.

[ref62] Ketter E. (2016). Destination image restoration on facebook: the case study of Nepal’s Gurkha earthquake. J. Hosp. Tour. Manag. 28, 66–72. doi: 10.1016/j.jhtm.2016.02.003

[ref63] Kim H. Richardson S. L. (2003). Motion picture impacts on destination images. Ann. Tour. Res. 30, 216–237. doi: 10.1016/S0160-7383(02)00062-2

[ref64] Kim J. Shinaprayoon T. Ahn S. J. (2022). Virtual Tours encourage intentions to travel and willingness to pay via spatial presence, enjoyment, and destination image. J. Curr. Issues Res. Advert. 43, 90–105. doi: 10.1080/10641734.2021.1962441

[ref65] King L. J. Golledge R. G. (1978). Cities, space, and behavior: the elements of urban geography, *Vol*. 393. Michigan: Prentice Hall, 236–256.

[ref66] Kitchin R. Blades M. (2002). The cognition of geographic space. I. B, Tauris: London, UK.

[ref67] Koch R. Latham A. (2017). Key thinkers on cities. NewYork: Sage.

[ref68] Kong D. (2020). Network media: a new way to reconstruct urban spatial image. Jiangxi Soc. Sci. 40, 240–270.

[ref69] Kordel S. (2016). The production of spaces of the ‘good life’ - the case of lifestyle migrants in Spain. Leis. Stud. 35, 129–140. doi: 10.1080/02614367.2014.962592

[ref70] Krupskyi O. P. Grynko T. (2018). Role of cognitive style of a manager in the development of tourism companies’ dynamic capabilities. Tour. Hosp. Manag. 24, 1–21. doi: 10.20867/thm.24.1.5

[ref71] Lalicic L. Marine-Roig E. Ferrer-Rosell B. Martin-Fuentes E. (2021). Destination image analytics for tourism design: an approach through Airbnb reviews. Ann. Tour. Res. 86:103100. doi: 10.1016/j.annals.2020.103100

[ref72] Lathia N. Quercia D. Crowcroft J. (2012). “The hidden image of the city: sensing community well-being from urban mobility.” in *International conference on pervasive computing*. Springer, Berlin, Heidelberg. 91–98.

[ref73] Lee C. K. Lee Y. K. Lee B. (2005). Korea’s destination image formed by the 2002 world cup. Ann. Tour. Res. 32, 839–858. doi: 10.1016/j.annals.2004.11.006

[ref74] Lee Y. Schmidt C. G. (1986). Urban spatial cognition: a case study of Guangzhou, China. Urban Geogr. 7, 397–412. doi: 10.2747/0272-3638.7.5.397

[ref75] Lefebvre H. (2003). The urban revolution. Minnesota: U of Minnesota Press, 35.

[ref76] Lefebvre H. (2012). “From the production of space” in Theatre and Performance Design. (London: Routledge), 81–84.

[ref77] Lei J. Zhao L. Chen D. (2020). Xi’an tourism analysis report. Am. J. Ind. Bus. Manag. 10, 1360–1367. doi: 10.4236/ajibm.2020.108090

[ref500] Li F. (2018). [@Fantsy Li]. [Video]. Available at: https://v.douyin.com/rQRQC94/ (Accessed November 19, 2018).

[ref501] Li F. (2022). [@cover News]. [Video]. Available at: https://v.douyin.com/rQRM4UN/ (Accessed May 30, 2022).

[ref78] Li H. Cui G. Zhou D. (2020). Urban space quality enhancement and feature preservation in the internet era. Land. Archit. Front. 8, 110–119. doi: 10.15302/J-LAF-1-030021

[ref79] Li Y. Guan M. Hammond P. Berrey L. E. (2021). Communicating COVID-19 information on Tik Tok: a content analysis of Tik Tok videos from official accounts featured in the COVID-19 information hub. Health Educ. Res. 36, 261–271. doi: 10.1093/her/cyab010, PMID: 33667311 PMC7989330

[ref80] Li X. Katsumata S. (2020). The impact of multidimensional country distances on consumption of specialty products: a case study of inbound tourists to Japan. J. Vacat. Mark. 26, 18–32. doi: 10.1177/1356766719842280

[ref81] Li J. Pearce P. L. Low D. (2018). Media representation of digital-free tourism: a critical discourse analysis. Tour. Manag. 69, 317–329. doi: 10.1016/j.tourman.2018.06.027

[ref82] Li X. Wirawan D. Li T. Yuan J. (2021). Behavioral changes of multichannel customers: their persistence and influencing factors. J. Retail. Consum. Serv. 58:102335. doi: 10.1016/j.jretconser.2020.102335

[ref83] Li Y. Xu X. Song B. He H. (2020). Impact of short food videos on the tourist destination image-take Chengdu as an example. Sustainability 12:6739. doi: 10.3390/su12176739

[ref84] Liu Y. Cheng T. (2020). Understanding public transit patterns with open geodemographics to facilitate public transport planning. Transp. Transp. Sci. 16, 76–103. doi: 10.1080/23249935.2018.1493549

[ref85] Liu Y. Zhang F. (2020). Mining urban perceptions from social media data. J. Spat. Inf. Sci. 20, 51–55. doi: 10.5311/JOSIS.2020.20.665

[ref86] Lu Y. (2018). The Association of Urban Greenness and Walking Behavior: using Google street view and deep learning techniques to estimate residents’ exposure to urban greenness. Int. J. Environ. Res. Public Health 15:1576. doi: 10.3390/ijerph15081576, PMID: 30044417 PMC6121356

[ref503] Lulu L. (2021). [@Sister Lu one meter seven 9 sets | Chongqing LiangRiYou visitors, handy. @ # trill little helper travel Chongqing tourism strategy # # # # big players]. [Video]. Available at: https://v.douyin.com/rQdLpsa/ (Accessed November 09, 2021).

[ref87] Lynch K. (1964). The image of the city. London: MIT Press, 71–79.

[ref88] Lynch K. S. (2022). Fans as transcultural gatekeepers: the hierarchy of BTS’ Anglophone Reddit fandom and the digital east-west media flow. New Media Soc. 24, 105–121. doi: 10.1177/1461444820962109

[ref89] Mac Kay K. J. Fesenmaier D. R. (2000). An exploration of cross-cultural destination image assessment. J. Travel Res. 38, 417–423. doi: 10.1177/004728750003800411

[ref90] Marengo D. Montag C. (2020). Digital Phenotyping of big five personality via Facebook data mining: a meta-analysis. Digit. Psychol. 1, 52–64. doi: 10.24989/dp.v1i1.1823

[ref91] Marques C. Vinhas da Silva R. Antova S. (2021). Image, satisfaction, destination and product post-visit behaviours: how do they relate in emerging destinations? Tour. Manag. 85:104293. doi: 10.1016/j.tourman.2021.104293

[ref92] Massood P. (2011). Black city cinema: African American urban experiences in film. Temple University Press.

[ref93] Medina Serrano J. C. Papakyriakopoulos O. Hegelich S. (2020). “Dancing to the partisan beat: a first analysis of political communication on Tik Tok.” in: *12th ACM conference on web science*. Southampton United Kingdom. 257–266.

[ref94] Milevsky Moshe A. (1997). Space-time diversification: which dimension is better? SSB. 07:97.

[ref95] Montag C. Lachmann B. Herrlich M. Zweig K. (2019). Addictive features of social media/messenger platforms and Freemium games against the background of psychological and economic theories. Int. J. Environ. Res. Public Health 16:2612. doi: 10.3390/ijerph16142612, PMID: 31340426 PMC6679162

[ref96] Mossberger K. Wu Y. Crawford J. (2013). Connecting citizens and local governments? Social media and interactivity in major U.S. cities. Gov. Inf. Q. 30, 351–358. doi: 10.1016/j.giq.2013.05.016

[ref97] Omar B. Dequan W. (2020). Watch, share or create: the influence of personality traits and user motivation on Tik Tok Mobile video usage. Int. J. Interact. Mob. Technol. 14:121. doi: 10.3991/ijim.v14i04.12429

[ref98] Oteros-Rozas E. Martín-López B. Fagerholm N. Bieling C. Plieninger T. (2018). Using social media photos to explore the relation between cultural ecosystem services and landscape features across five European sites. Ecol. Indic. 94, 74–86. doi: 10.1016/j.ecolind.2017.02.009

[ref99] Pacione M. (2009). Urban geography: A global perspective. 3rd Edn. London: Routledge.

[ref100] Pearce P. L. (1997). Mental souvenirs: a study of tourists and their city maps. Aust. J. Psychol. 29, 203–210. doi: 10.1080/00049537708255282

[ref101] Pearce D. G. (1998). Tourist districts in Paris: structure and functions. Tour. Manag. 19, 49–65. doi: 10.1016/S0261-5177(97)00095-2

[ref102] Pearce D. G. (1999). Tourism in Paris studies at the microscale. Ann. Tour. Res. 26, 77–97. doi: 10.1016/S0160-7383(98)00051-6

[ref103] Peel D. Lloyd G. (2008). New communicative challenges: Dundee, place branding and the reconstruction of a city image. Town Plan. Rev. 79, 507–532. doi: 10.3828/tpr.79.5.4

[ref104] Peng X. Bao Y. Huang Z. (2020). Perceiving Beijing’s “City image” across different groups based on Geotagged social media data. IEEE Access 8, 93868–93881. doi: 10.1109/ACCESS.2020.2995066

[ref105] Pike S. (2002). Destination image analysis—a review of 142 papers from 1973 to 2000. Tour. Manag. 23, 541–549. doi: 10.1016/S0261-5177(02)00005-5

[ref106] Pikkemaat B. (2004). The measurement of destination image: the case of Austria. Poznan Univ. Eco. Rev. 4, 87–102.

[ref107] Pop R.-A. Săplăcan Z. Dabija D.-C. Alt M.-A. (2022). The impact of social media influencers on travel decisions: the role of trust in consumer decision journey. Curr. Issue Tour. 25, 823–843. doi: 10.1080/13683500.2021.1895729

[ref108] Prayag G. Hosany S. Muskat B. Del Chiappa G. (2017). Understanding the relationships between tourists’ emotional experiences, perceived overall image, satisfaction, and intention to recommend. J. Travel Res. 56, 41–54. doi: 10.1177/0047287515620567

[ref109] Ramkissoon H. Nunkoo R. (2011). City image and perceived tourism impact: evidence from Port Louis, Mauritius. Int. J. Hosp. Tour. Adm. 12, 123–143. doi: 10.1080/15256480.2011.564493

[ref110] Rapoport A. (2016). Human aspects of urban form: Towards a man—Environment approach to urban form and design. Oxford: Elsevier.

[ref111] Salas-Olmedo M. H. Moya-Gómez B. García-Palomares J. C. Gutiérrez J. (2018). Tourists’ digital footprint in cities: comparing big data sources. Tour. Manag. 66, 13–25. doi: 10.1016/j.tourman.2017.11.001

[ref112] Sevin H. E. (2014). Understanding cities through city brands: City branding as a social and semantic network. Cities 38, 47–56. doi: 10.1016/j.cities.2014.01.003

[ref113] Shao J. Lee S. (2020). The effect of Chinese adolescents’ motivation to use Tiktok on satisfaction and continuous use intention. J. Converg. Cult. Technol. 6, 107–115. doi: 10.17703/JCCT.2020.6.2.107

[ref114] Shao J. Li X. Morrison A. M. Wu B. (2016). Social media micro-film marketing by Chinese destinations: the case of Shaoxing. Tour. Manag. 54, 439–451. doi: 10.1016/j.tourman.2015.12.013

[ref115] Shao T. Wang R. Hao J. X. (2019). “Visual destination images in user-generated short videos: an exploratory study on Douyin.” in *2019 16th international conference on service systems and service management (ICSSSM). IEEE, Shenzhen, China*. 1–5.

[ref116] Shen Y. (2019). How net red cities stay red. People’s Trib. 30, 130–131.

[ref117] Shi Y. (2021). Research on urban tourism communication strategy of douyin short video. OALib. 08, 1–5. doi: 10.4236/oalib.1107061

[ref118] Shoval N. (2018). Urban planning and tourism in European cities. Tour. Geogr. 20, 371–376. doi: 10.1080/14616688.2018.1457078

[ref119] Sidorenko-Bautista P. Herranz de la Casa J. M. Cantero de Julián J. I. (2021). Use of new narratives for COVID-19 reporting: from 360o videos to ephemeral Tik Tok videos in online media. Tripodos 1, 105–122. doi: 10.51698/tripodos.2020.47p105-122

[ref120] Smith A. (2005). Reimaging the city. Ann. Tour. Res. 32, 217–236. doi: 10.1016/j.annals.2004.07.007

[ref121] Smith A. McGillivray D. (2022). The long-term implications of mega-event projects for urban public spaces. Sport Soc. 25, 2107–2123. doi: 10.1080/17430437.2020.1826934

[ref122] Son A. (2005). The measurement of tourist destination image: applying a sketch map technique. Int. J. Tour. Res. 7, 279–294. doi: 10.1002/jtr.532

[ref123] Song Y. (2022). “Research on urban brand promotion based on short video marketing in the new media environment: taking the spring city of jinan as an example.” in *Presented at the 2021 international conference on social development and media communication (SDMC 2021)*. Atlantis Press, 1467–1472.

[ref124] Stankov U. Lazić L. Dragićević V. (2010). The extent of use of basic Facebook user-generated content by the national tourism organizations in Europe. EJTR 3, 105–113. doi: 10.54055/ejtr.v3i2.51

[ref125] Stepchenkova S. Eales J. S. (2011). Destination image as quantified media messages: the effect of news on tourism demand. J. Travel Res. 50, 198–212. doi: 10.1177/0047287510362780

[ref126] Stephanson A. Jameson F. (1989). Regarding postmodernism: a conversation with fredric jameson. Soc. Text. 3, 3–44. doi: 10.2307/827806

[ref127] Su J. (2020a). “Image reproduction and expression reconstruction: Urban image communication in the era of short video.” in *Proceedings of the 7th international conference on education, language, art and inter-cultural communication (ICELAIC 2020)*. pp. 428–431. Atlantis Press.

[ref128] Su J. (2020b). “Construction and dissemination of city image in mobile short videos.” in *4th international conference on culture, education and economic development of modern society (ICCESE 2020)*. Atlantis Press, 531–534.

[ref129] Sugandini D. (2020). Hasil cek plagiasi perceived value, eword-of-mouth, traditional word-of-mouth, and perceived quality to destination image of vacation tourists. Available at: http://eprints.upnyk.ac.id/id/eprint/23040 (Accessed November 10, 2022).

[ref130] Sun W. (2018). Research approaches to and theoretical innovation for urban communication. Mod. Commun. 40, 29–40.

[ref131] Szubert M. Warcholik W. Żemła M. (2021). The influence of elements of cultural heritage on the image of destinations, using four polish cities as an example. Land 10:671. doi: 10.3390/land10070671

[ref132] Takeuchi Y. Perlin K. (2012). “Clay vision: the (elastic) image of the city.” in *Proceedings of the SIGCHI conference on human factors in computing systems, CHI’12*. Association for Computing Machinery, New York, NY, USA. 2411–2420.

[ref133] Tasci A. D. A. Uslu A. Stylidis D. Woosnam K. M. (2022). Place-oriented or people-oriented concepts for destination loyalty: destination image and place attachment versus perceived distances and emotional solidarity. J. Travel Res. 61, 430–453. doi: 10.1177/0047287520982377

[ref134] Tavitiyaman P. Qu H. Tsang W. L. Lam C. R. (2021). The influence of smart tourism applications on perceived destination image and behavioral intention: the moderating role of information search behavior. J. Hosp. Tour. Manag. 46, 476–487. doi: 10.1016/j.jhtm.2021.02.003

[ref135] TikTok Headline Index, City Branding Research Office, Tsinghua University (2018). Shake out. White Paper on Short Video and City Image Research, September.

[ref136] Tussyadiah I. P. Fesenmaier D. R. (2009). Mediating tourist experiences. Ann. Tour. Res. 36, 24–40. doi: 10.1016/j.annals.2008.10.001

[ref137] Uusitalo M. (2014). “Differences in tourists’ and local residents’ perceptions of tourism landscapes: a case study from Ylläs, Finnish Lapland” in Frontiers in nature-based tourism. (London: Routledge), 144–167.

[ref138] Vaez S. Burke M. Yu R. (2020). Visitors’ wayfinding strategies and navigational aids in unfamiliar urban environment. Tour. Geogr. 22, 832–847. doi: 10.1080/14616688.2019.1696883

[ref139] Walmsley D. J. Jenkins J. M. (1992). Tourism cognitive mapping of unfamiliar environments. Ann. Tour. Res. 19, 268–286. doi: 10.1016/0160-7383(92)90081-Y

[ref140] Wang Y. Feng D. (2021). History, modernity, and city branding in China: a multimodal critical discourse analysis of Xi’an’s promotional videos on social media. Soc. Semiot., 1–24. doi: 10.1080/10350330.2020.1870405

[ref141] Wang Y. Xu R. (2021). The generation of net red of punch card attractions: analysis of users’ daily practice based on short video environment. Chin. You. Stu. 02, 105–112. doi: 10.19633/j.cnki.11-2579/d.2021.0046

[ref142] Weijs-Perrée M. Dane G. van den Berg P. (2020). Analyzing the relationships between citizens’ emotions and their momentary satisfaction in urban public spaces. Sustainability 12:7921. doi: 10.3390/su12197921

[ref143] Wen H. Josiam B. M. Spears D. L. Yang Y. (2018). Influence of movies and television on Chinese tourists perception toward international tourism destinations. Tour. Manag. Perspect. 28, 211–219. doi: 10.1016/j.tmp.2018.09.006

[ref144] Wengel Y. Ma L. Ma Y. Apollo M. Maciuk K. Ashton A. S. (2022). The Tik Tok effect on destination development: famous overnight, now what? J. Outdoor Recreat. Tour. 37:100458. doi: 10.1016/j.jort.2021.100458

[ref145] Wu X. L. (2011). Western space production theory and our historical choice of space production. South. Acad. Res. 7, 19–25. doi: 10.13658/j.cnki.sar.2011.06.014

[ref146] Wu Z. C. Zhang L. Y. Zheng Z. Q. (2019). A study of the spatial production of tourist communities in ancient cities in the tourism field - based on the perspective of Levi Vogel’s theory of spatial production. J. Tour. 12, 86–97.

[ref147] Xia J. Arrowsmith C. Jackson M. Cartwright W. (2008). The wayfinding process relationships between decision-making and landmark utility. Tour. Manag. 29, 445–457. doi: 10.1016/j.tourman.2007.05.010

[ref148] Yang J. Zhang D. Liu X. Hua C. Li Z. (2022). Destination endorsers raising on short-form travel videos: self-image construction and endorsement effect measurement. J. Hosp. Tour. Manag. 52, 101–112. doi: 10.1016/j.jhtm.2022.06.003

[ref149] Ye C. Zhang F. Mu L. Gao Y. Liu Y. (2021). Urban function recognition by integrating social media and street-level imagery. Environ. Plan. B Urban Anal. City Sci. 48, 1430–1444. doi: 10.1177/2399808320935467

[ref150] Young M. (1999). Cognitive maps of nature-based tourists. Ann. Tour. Res. 26, 817–839. doi: 10.1016/S0160-7383(99)00023-7

[ref151] Zhang Z. H. Wang L. Zhang P. (2009). A study on tourism destination image with place theory from abroad. Mod. Urban Res. 5, 69–75.

[ref152] Zhao Z. Ren J. Wen Y. (2020). Spatial perception of urban forests by citizens based on semantic differences and cognitive maps. Forests 11:64. doi: 10.3390/f11010064

[ref504] Zhihong X. (2020). [@Big bear and Ball ball]. [Video]. Available at: https://v.douyin.com/rQddmyD/ (Accessed December 19, 2020).

[ref505] Zhihong X. (2021). [@Big bear and Ball ball]. [Video]. Available at: https://v.douyin.com/rQdS2mk/ (Accessed June 06, 2021).

[ref153] Zhou W. Pickett S. T. A. Cadenasso M. L. (2017). Shifting concepts of urban spatial heterogeneity and their implications for sustainability. Landsc. Ecol. 32, 15–30. doi: 10.1007/s10980-016-0432-4

[ref154] Zhou L. J. Wang T. (2014). Social media: a new vehicle for city marketing in China. Cities 37, 27–32. doi: 10.1016/j.cities.2013.11.006

[ref506] Zihan Z. (2019). [@What to eat today]. [Video]. Available at: https://v.douyin.com/rQdnxCF/ (Accessed November 09, 2019).

