# Supplementary material for: Reproduction, cultural symbolism, and online relationship: Constructing city spatial imagery on TikTok
Source: Front Psychol. 2022 Dec 23;13:1080090. doi: 10.3389/fpsyg.2022.1080090 (PMC9928210; doi:10.3389/fpsyg.2022.1080090)
Supplement: Supplementary file 1 [file Data_Sheet_1.docx]

**Pictures in appendix:**

Picture 1


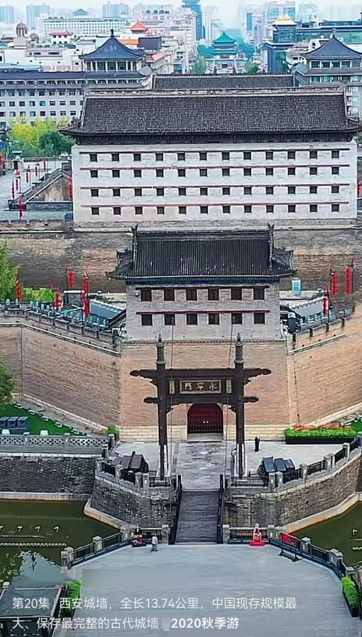


Picture 1: Xi'an City Wall

Picture source: Peng, Zhou, [@Aerial Photo of China] (2020, October 19). [video]. <https://v.douyin.com/rp4of8h/>. Reproduced with permission.

Translation: Xi 'an city wall, 13.74 km, China's largest and most complete existing ancient city wall # 2020 fall tour

Picture 2


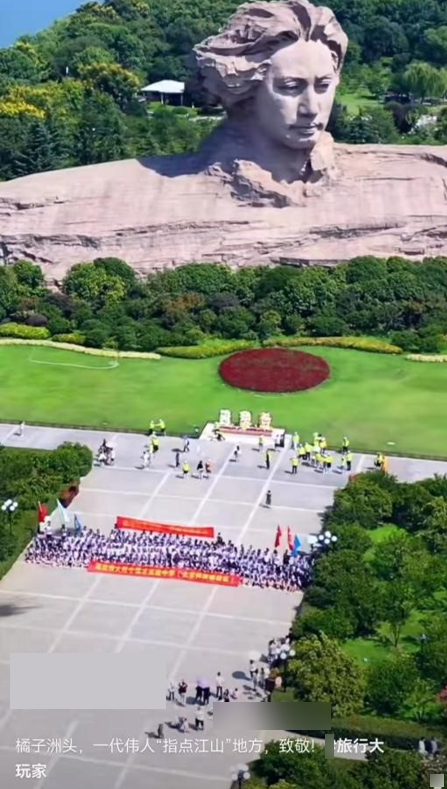


Picture 2: Orange Island

Picture source: Zifeng, Liu, @Fengge Travel [Record of China] (2022, August 15). [video]. <https://v.douyin.com/rpqwk3f/>. Reproduced with permission.

Translation: Changsha Orange Island head, a generation of great people "guide the river and mountains" place, salute! Really big player # # travel Changsha summer travel more # compass

Picture 3


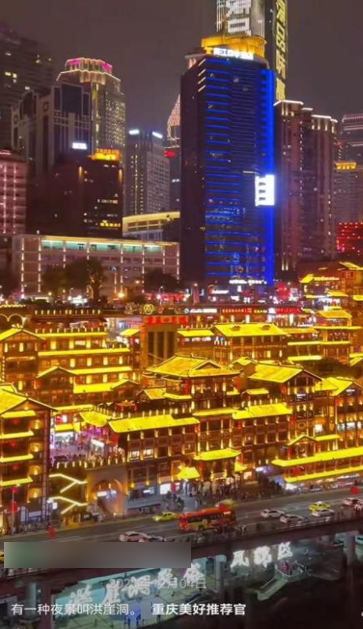


Picture 3: Hongya Cave

Picture source: Huimin, Chen, [@Shancheng Miss Chen] (2021, December 04). [video]. <https://v.douyin.com/rpbBXXv/>. Reproduced with permission.

Translation: There is a night scene called Hongya Cave. # chongqing good recommend officer

Picture4


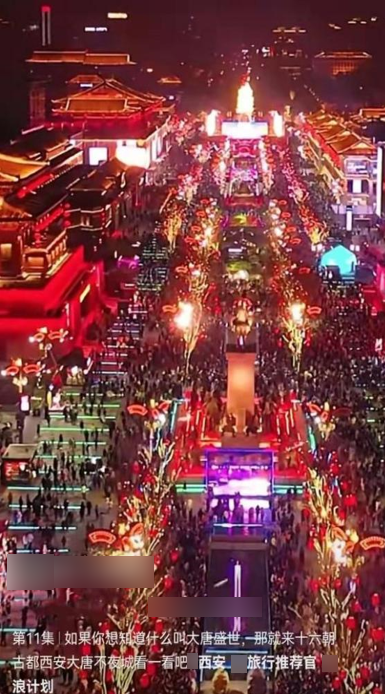


Picture 4: Datang City of Night

Picture source: Zixiao, Yuan, [@Xiao Yuanbao Resignation Travel Story] (2020, November 06). [video]. HTTP: / / <https://v.douyin.com/rpb2qBd/>. Reproduced with permission.

Translation: If you want to know what call Datangshengshi, it would be to have a look ten dynasties ancient capital xi 'an Datang city that never sleeps # xi 'an # # travel recommends officer wave plan

Picture 5


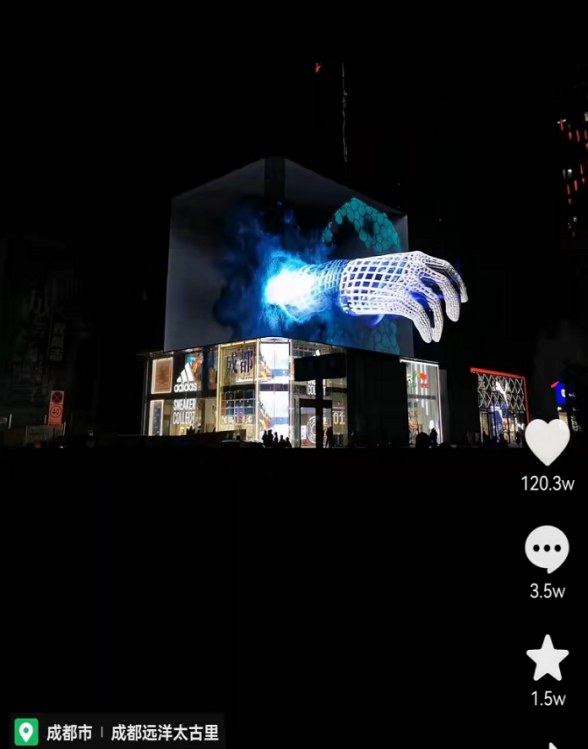


Picture 5: Taikoo Li naked eye 3D screen

Picture source: Hongbo, Chen, [@_ Chen Baibai] (2020, November 06). Chengdu Taikoo Li naked eye 3D. # noise shielding technique [video]. <https://v.douyin.com/rpbC1TK/>. Reproduced with permission.

Picture 6


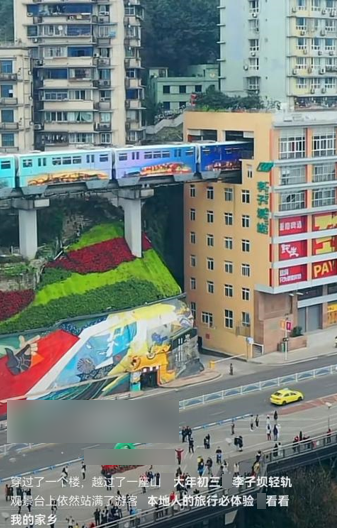


Picture 6: The Li Ziba light rail tram

Picture source: Jiawen, Xu, [@Wudu Chongqing] (2021, December 04). [video]. <https://v.douyin.com/rpbp4JW/>. Reproduced with permission.

Translation: Through a floor, across a mountain light rail observation deck # # annual grade plum pa still filled the tourists travel will experience # # the natives to see my hometown

Picture 7


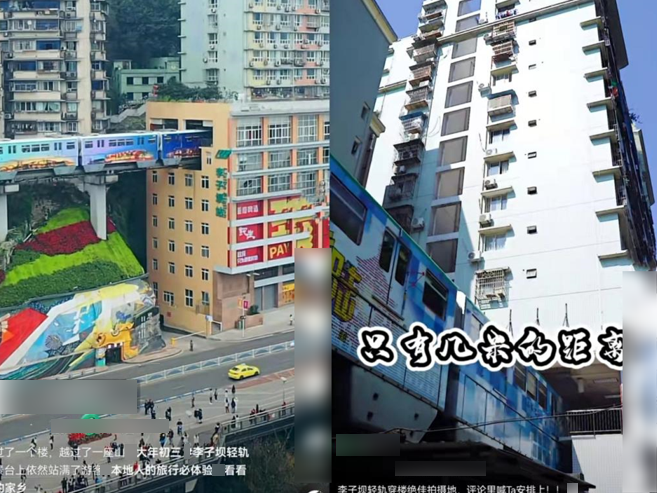


Picture 7: Liziba Metro Station

Picture source: Yongqiang, Du, [@dog brother] (2020, September 09). [video]. <https://v.douyin.com/rpgy8o6/>. Reproduced with permission.

Translation: Excellent shooting spot of the Lee Dam Light Rail crossing building, let Ta arrange it in the comments!! # # Chongqing light rail trill shout you to accept # web celebrity clock PM @ trill food

Picture 7 on the left is the same as picture 6, while the picture on the right shows the addition of a viewing platform at Liziba, with the video captioned "Only a few metres away" and "Excellent place to shoot the LRT through the building at Liziba, ask your friends in the comments to arrange it!

Picture 8


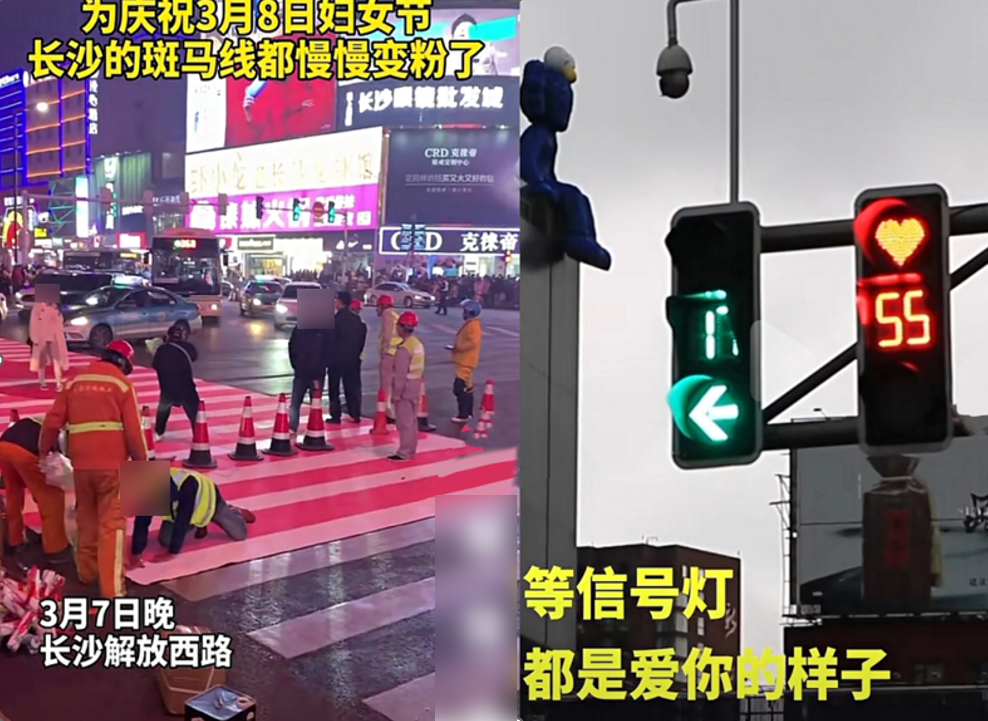


Picture 8: Jiefang West Road and a signal in Changsha

Picture source: Bin, Fang, [@Xiaofang Student] (March 08, 2021). [video]. <https://v.douyin.com/rpgfxUw/>. Reproduced with permission.

Translation: Changsha send you a heart look, Changsha 51 business circle, to celebrate 3.8 women's Day, pink zebra romantic line!

Picture 9


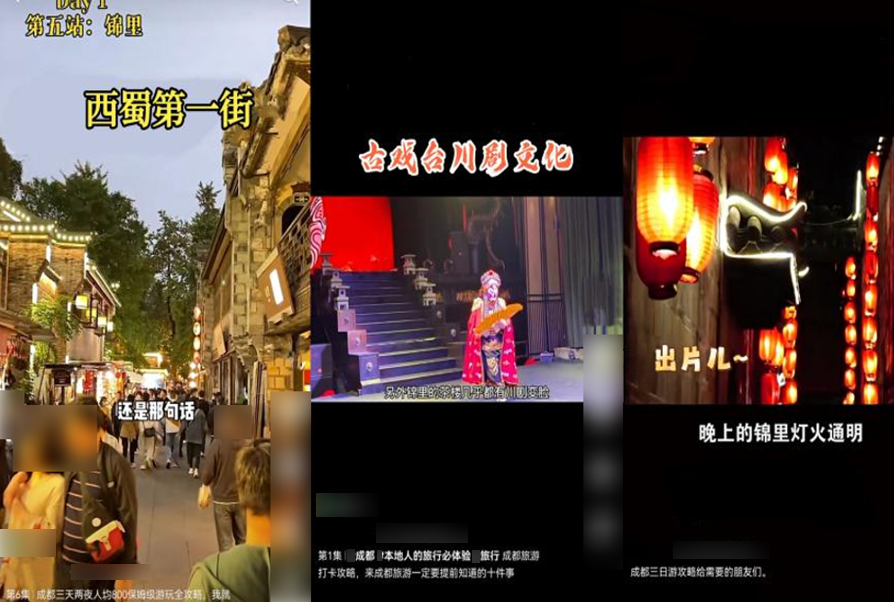


Picture 9: Jinli in Chengdu

Left picture source: Qiang, Chen, [@Chen Baobao] (2021, February 03). [video]. https://v.douyin.com/rpgXDUK/

Translation: Clock in Chengdu tourism strategy, to Chengdu tourism must know in advance of 10 things # # # Chengdu local travel will experience travel

Right two pictures source: Qiang, Chen, [@Chen Baobao] (2019, November 29). [video]. <https://v.douyin.com/rpg3CLb/>. Reproduced with permission.

Translation: Three-day Tour guide to Chengdu for friends in need.

The text of the left picture is "The First Street of Western Sichuan", the text of the middle picture is "Sichuan Opera Culture in the Ancient Theatre", and the right picture shows "Out of the picture~, Jinli is brightly lit up at night".

Picture 10


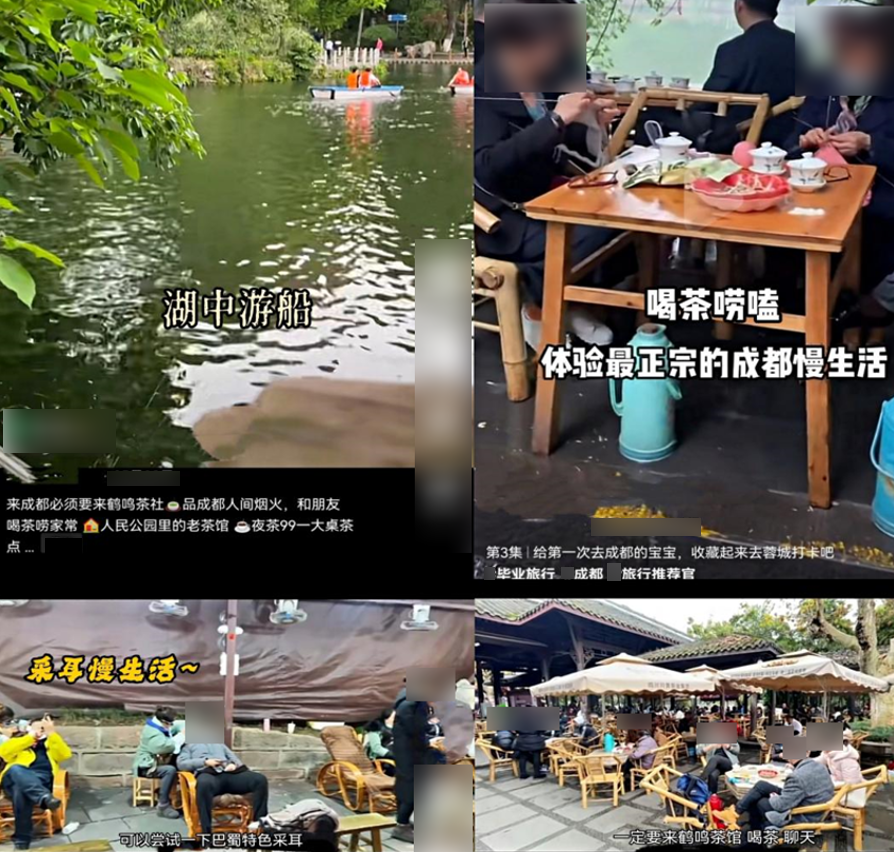


Picture 10: Life in Chengdu

Picture source: Liya, Liu, [@Tiantian Eclipse Story] (2022, May 09). [video]. <https://v.douyin.com/rpgcNWp/>. Reproduced with permission.

Translation: To come to Chengdu, you must come to the Heming Teahouse 🍵 to taste the Chengdu human fireworks, drink tea with friends and chat home 🏠 the old teahouse in the People's Park ☕️ night tea 99 A large table of tea 📷 recently there are tea art performances in the evening, Local treasure foods during the day is also a piece of # # # Chengdu gourmet trill food recommendation officer

The video captioned above right is about drinking tea and chattering, experiencing the most authentic Chengdu slow life. The text is "For first time visitors, bookmark your trip to Chengdu", #graduation trip, Chengdu #Travel recommendation officer.

The video caption on the bottom left shows the slow life of ear picking. The text is “You can try ear picking, a specialty of Sichuan”.

The video caption on the bottom right is "Be sure to come to Heming Tea House for tea and conversation.

Picture 11


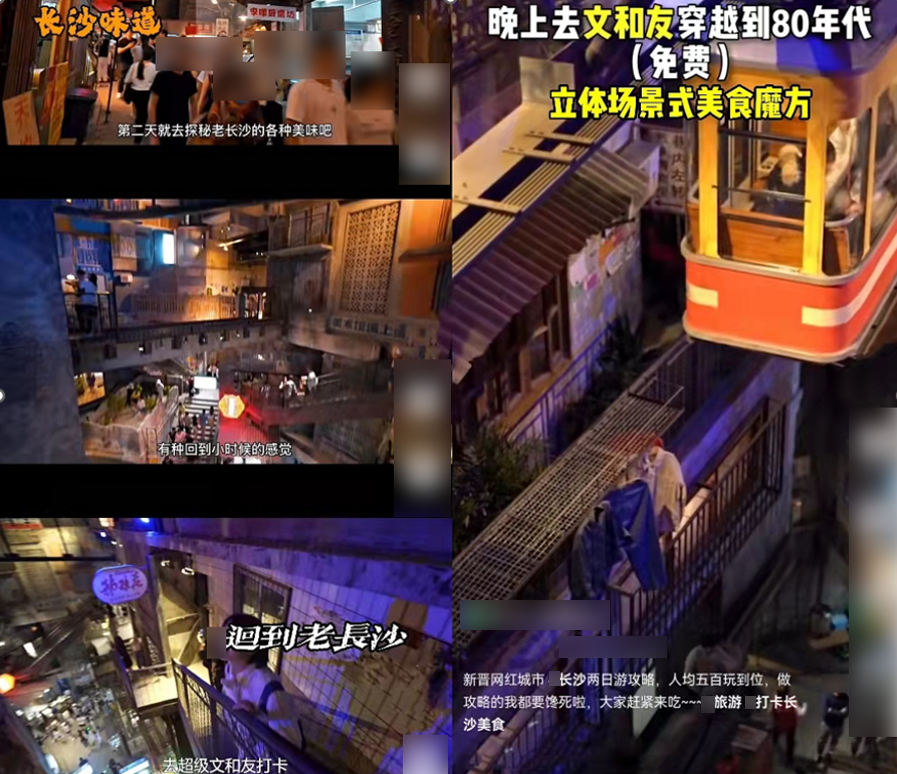


Picture 11: Changsha Super Wenheyou

Picture source: Peichun, Zhang, [@Call me Travel bug] (2020, December 08). [video]. <https://v.douyin.com/rpp3Axt/>. Reproduced with permission.

Translation: New web celebrity city changsha LiangRiYou strategy, five hundred per head to play in place, do I have to chan strategy is dead, you hurriedly to eat ~ ~ ~ # # travel clock in changsha food

The video caption on the top left is “Taste of Changsha, let's explore the various delicacies of Changsha”.

In the middle left is a childhood kiosk at Super Wenheyou in Changsha with the caption "It feels like going back to my childhood"

The bottom left is the caption "Go back to old Changsha and hit Super Wenheyou.

On the right, the caption reads "Travel back to the 80s at night at Super Wenheyou (free). #Changsha #travel # playing cards Changsha food

Picture 12 are some comments of the video in picture 11, For copyright reasons, only translations are shown here:

Comment 1: Super Wenheyou! It's like being a kid again

Comment 2: Super Wenheyou, occasionally visit, feel like back to the 80's and 90's!

Comment 3: You take me back to my childhood, the standard post-80s generation who grew up in a small city

Comment 4: This musical moment takes me back to my childhood

Picture 12: Nan, Wang, "wild Wang Xiaotu @" (2021, April 24). 3 days and 2 nights | changsha strategy - 1000 pieces per capita, the text version in the end. # ticket blind box Offer first class # 98 yuan ticket blind box # # clock in changsha tourism in changsha good [video]. https://v.douyin.com/rppwnR4/

Picture 13


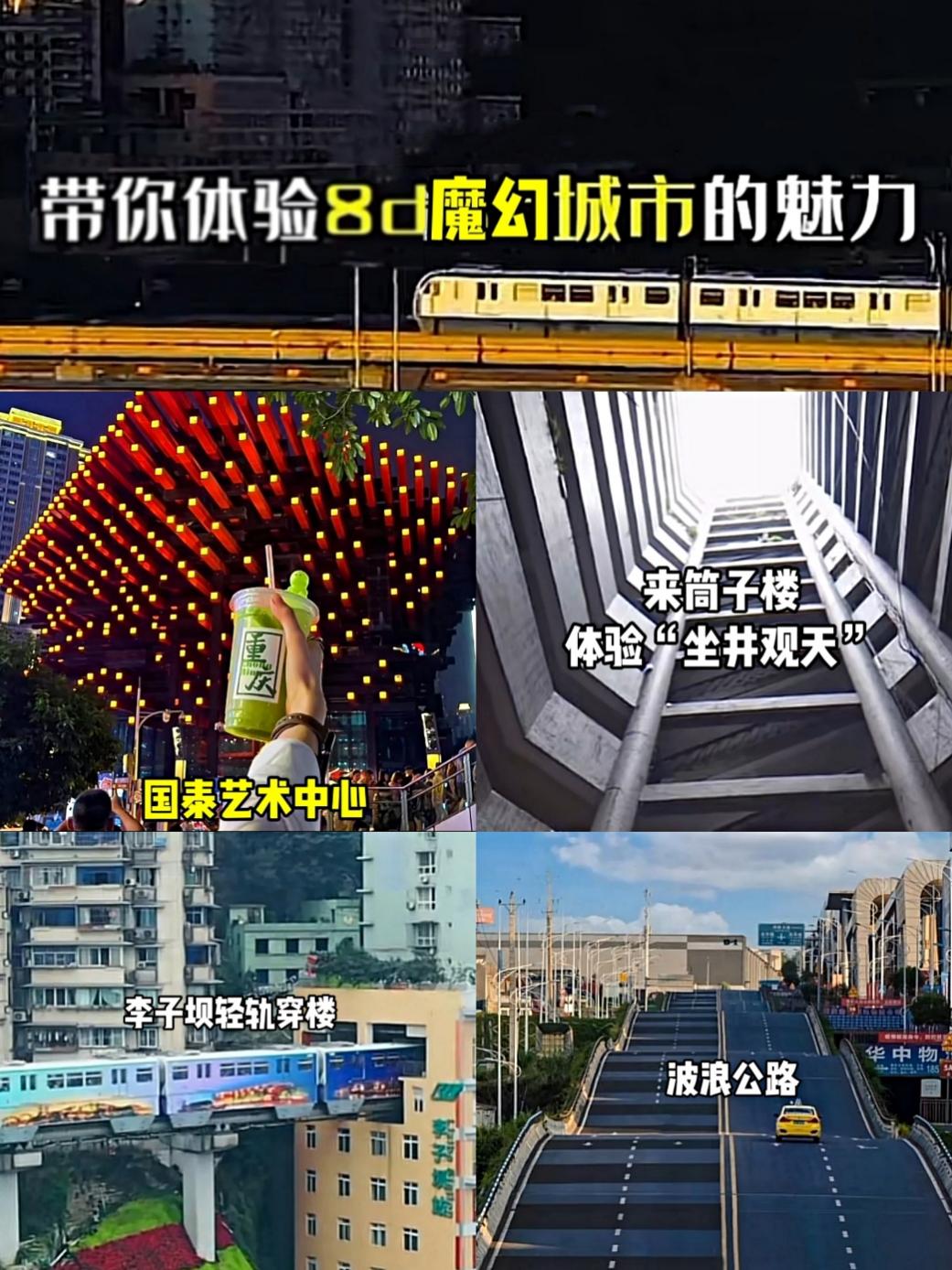


Picture 13: 8D Magic Space in Chongqing

Picture source: Li, Fan, @Fantsy Li (October 23, 2022). [video]. <https://v.douyin.com/rpsetev/>. Reproduced with permission.

Translation: Wavy Chongqing highway, driving, such as on a roller coaster # Chongqing

The top left picture is the Cathay Art Centre, the top right picture is the building of the special structure in Chongqing, the caption reads "Come to the silo and experience sitting on the well", the bottom left picture is the caption reads Li Ziba Light Rail through the building, the bottom right picture is the wave-liked highway.

Picture 14


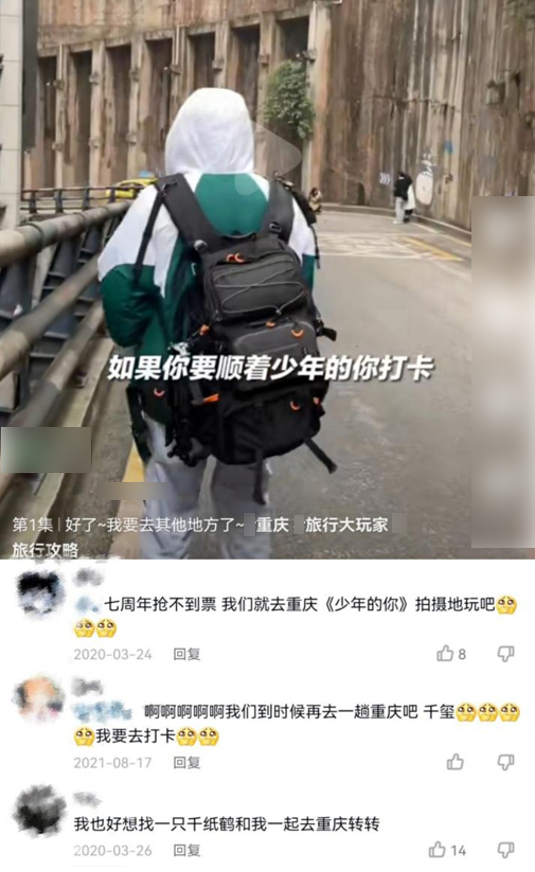


Picture 14: Filming location of the film "Teenage You"

Picture source: Yun, Zhao, [@Bad cat] (2022, January 29). [video]. <https://v.douyin.com/rpsRnUs/>. Reproduced with permission.

Translation: Ok ~ I will go to other places the big player # ~ # # Chongqing travel; travel guide

Picture 15


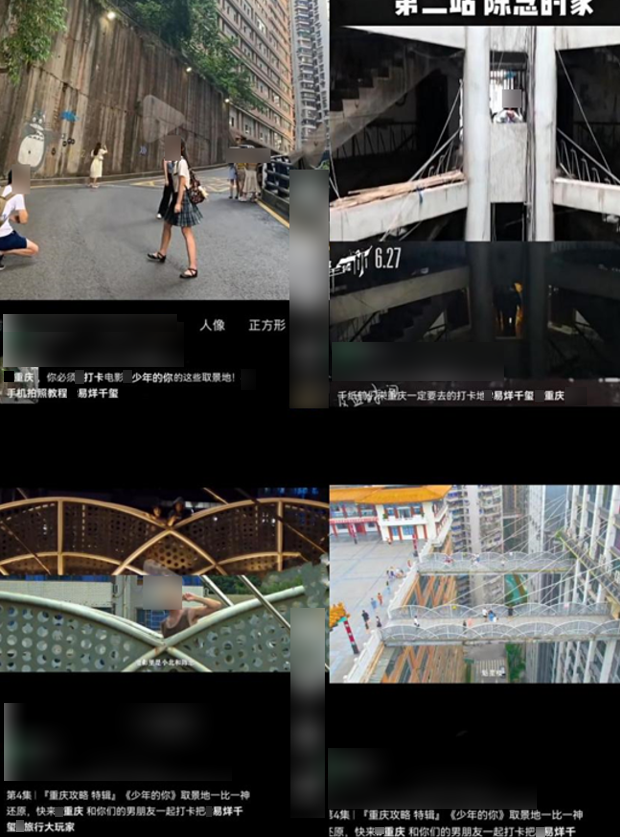


Picture 15: Fans going to the film's filming location

Picture source: Yayan, Wu, [@Cosmos Yanyan] (July 19, 2021). [video]. <https://v.douyin.com/rpsd2kC/>"Chongqing Introduction Special" "Young You" the scene of a one-by-one god restoration, Come on # clock in Chongqing and your boyfriend to # # Jackson travel big players. Reproduced with permission.

Picture source: Yayan, Wu, [@Cosmos Yanyan] (2020, August 24). [video]. https://v.douyin.com/rpsytSG/ # Chongqing, you must # punch the movie # teen for your location! # # phone photo tutorial Jackson. Reproduced with permission.

Picture source: Yayan, Wu, [@Cosmos Yanyan] (2019, October 31) [video]. <https://v.douyin.com/rpstXhd/> papercranes to clock in Chongqing must go to # # # Jackson Chongqing youth you @ trill little helper Reproduced with permission.

Translation: Above left is the Railway High School in Chongqing, where the video shows a photo shoot instruction. The theme is "You must clock these locations for the film Teenage You" # check in, # Teenage You # a mobile phone photo tutorial #Yi Yang Qianxi (the main actor of the film). The top right picture is the Kui Xing Building, the caption is "The second stop, the home of Chen Nian (the name of the film's main character)", the text is “a paper crane (the fan name them as paper crane) in Chongqing must visit this place”, #Yi Yang Qianxi# Chongqing. The bottom left picture is the Crown Escalator and the bottom right picture is Zhongshan 4th Road. The places in the picture are all shooting places of the film “Teenage You”.

Picture 16


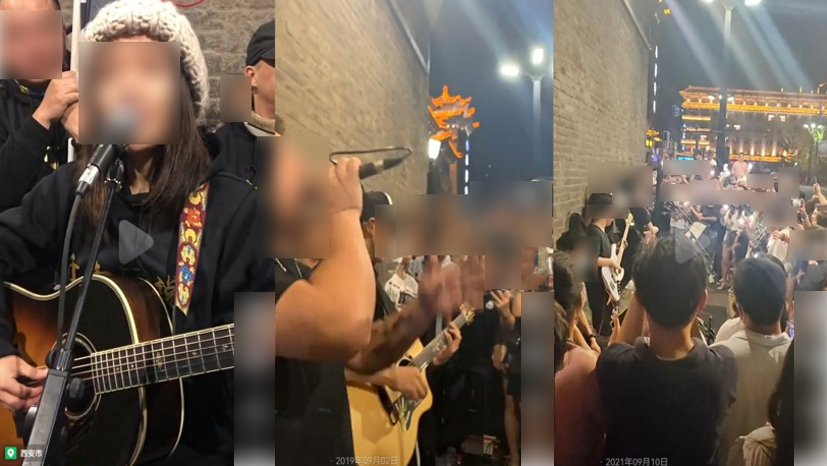


Picture 16: Folk singers singing in the streets of Xi'an at the South Gate under the city walls

Picture source: Shi, Zhang, [@ Listen to Nanmen] (2019, September 02). [video]. <https://v.douyin.com/rpsCGgQ/>. Reproduced with permission.

Translation: Xi 'an city wall under the "hua hin old cavity # listen to south gate release @ @ xi 'an xian wen brigade voices @ Hao Haohan @ zhang qiang MusicMan
